# 

**Published:** 1930

**Authors:** 


Vol. XLVII. No. 176. SUMMER, 1930.

### The Bristol Medico-Chirurgical Journal

Library of Cong[illegible] ... [illegible] Deposit

PUBLISHED UNDER THE AUSPICES OF

THE BRISTOL MEDICO-CHIRURGICAL SOCIETY.

*Editor:* J. A. NIXON, C.M.G., M.D., F.R.C.P.

WITH WHOM ARE ASSOCIATED

E. W. HEY GROVES, M.S., F.R.C.S., B.Sc.
E. WATSON-WILLIAMS, M.C., Ch.M., F.R.C.S.E.,
A. E. ILES, O.B.E., M.B., B.S. Lond., F.R.C.S.

CAREY F. COOMBS, M.D., F.R.C.P., *Assistant Editor.*

*Editorial Secretary:* A. L. FLEMMING, M.B., Ch.B.

BRISTOL: J. W. ARROWSMITH LTD., 11 QUAY STREET.
London: J. W. Arrowsmith (London) Ltd., 57 Gower Street, W.C.

Price Three Shillings net.

